# Pain and infection are the primary reasons for elective retrieval of bone-anchored implants

**DOI:** 10.1007/s00405-026-10072-8

**Published:** 2026-03-14

**Authors:** Marsel Ganeyev, Heithem Ben Amara, Peter Monksfield, Myrthe Hol, Robert J. Stokroos, Malou Hultcrantz, Howard Savage Jones, Margarita Trobos, Peter Thomsen, Anders Palmquist, Martin L. Johansson

**Affiliations:** 1 Department of Biomaterials, Institute of Clinical Sciences, Sahlgrenska Academy, University of Gothenburg, P.O. Box 412, Gothenburg, 405 30 Sweden; 2 Oticon Medical AB, Research & Technology, Askim, Sweden; 3 University Hospitals Birmingham, Birmingham, UK; 4 University Medical Center Utrecht, Utrecht, The Netherlands; 5 University Medical Center Groningen, Groningen, The Netherlands; 6 Institution CLINTEC, Karolinska Institutet, Stockholm, Sweden; 7 Midland Regional Hospital, Tullamore, Ireland; 8 Department of Biomedical Dental Sciences, College of Dentistry, Imam Abdulrahman Bin Faisal University, Dammam, Saudi Arabia

**Keywords:** Percutaneous implant, Bone-anchored implant, Infection, Inflammation, Pain

## Abstract

**Purpose:**

Elective removal of bone-anchored hearing implants and epistheses is uncommon, limiting the understanding of the biological factors contributing to adverse reactions, implant success or failure. Therefore, the tissue-level response at the implant interface remains poorly understood.

**Methods:**

This study examined 21 explanted bone-anchored implants and interfacial tissues from 19 patients using a controlled retrieval protocol. The clinical data were correlated with histological, immunohistochemical, microbiological, and structural analyses.

**Results:**

Pain (12/19) and infection (7/19) were the most common reasons for elective implant retrieval and in 5 cases occurred together. All explants, apart from one, showed significant osseointegration and maintained stability in situ, independent of their design characteristics. *Staphylococcus aureus* was isolated in five patients—four of whom reported pain even without overt infection. *Staphylococcus epidermidis* was detected in two patients: one with pain and infection and the other asymptomatic with a resolved infection. *S. aureus* colonization was associated with intense inflammation, marked by the presence of neutrophils, iNOS+ macrophages, CD3 + T-cells, and CD20 + B-cells.

**Conclusion:**

Pain and infection—often involving *Staphylococcus* species—are key drivers of implant retrieval. These findings emphasize the complex immune response at the implant site and the diagnostic value of analysing electively retrieved implants for improving patient outcomes.

**Supplementary Information:**

The online version contains supplementary material available at 10.1007/s00405-026-10072-8.

## Introduction

Bone-anchored implants rely on osseointegration, which is defined as a direct structural and functional connection between living bone and the surface of a load-bearing implant, for their retention and functionality [1]. Despite their widespread clinical use, the existing knowledge on implant performance is derived primarily from clinical assessments, such as patient-reported symptoms and functional outcomes [2, 3]. In contrast, only a few studies have examined the tissue‒implant interface in situ or retrieved via histological, cellular, or microbiological analyses [4–10]. Thus, as elective retrievals are rare, the mechanisms underlying implant success or failure remain poorly understood at the cellular and tissue levels [11].

Research examining explanted implants has reported variable bone‒implant contact, soft tissue inflammation, and immune cell infiltration in cases of failed osseointegration or chronic pain [4, 8, 9, 11–14]. However, clear correlations between such findings and patient symptoms have yet to be established. This underscores the need for more structured approaches to retrieval, sampling, and analysis. Retrieved and adequately preserved implants thus represent a valuable, underutilized resource for advancing our understanding of the biological signatures associated with clinical outcomes.

The percutaneous bone-anchored hearing system (BAHS) was introduced in the late 1970 s and consists of a sound processor connected to a skin-penetrating abutment mounted on a threaded titanium implant that is inserted into the temporal bone in a retroauricular position. The treatment is indicated for patients with mixed and conductive hearing loss or single-sided deafness. Since its introduction, BAHS have undergone significant developments in terms of design, surgical technique, and patient selection. Implant geometry has evolved from narrow Ø3.75 mm medical-grade pure titanium fixtures with minimally rough, machined surfaces to wider Ø4.5 mm implants featuring surface modifications such as blasting (TiOblast™, BI300, Cochlear AB, Mölnlycke, Sweden) [15, 16] and laser-ablation (Ponto BHX, Oticon Medical AB, Askim, Sweden) [17, 18], both aimed at improving osseointegration. The abutments have also evolved, including hydroxyapatite-coated types (BIA400) [19] and machined variants with distinct surface finishes [20] to optimize soft tissue response and hygiene. In parallel, surgical techniques have shifted from tissue reduction to tissue preservation and minimally invasive methods such as the minimally invasive Ponto procedures (MIPS and MONO), shortening the implantation procedure and improving clinical outcomes [21, 22].

Apart from audiological performance, BAHS has been proven to significantly improve the patients’ quality of life [23] with a reported implant survival rate above 92% [24]. However, similar to other percutaneous implants, complications such as adverse soft tissue reactions [23], mechanical instability [25], and chronic pain [9, 25–27] occur in a subset of patients, sometimes necessitating surgical revision or retrieval of the implant.

Previously, in a newly started implant retrieval network (IRN), our group conducted a detailed case study analysis on a patient with recurrent adverse soft tissue reactions and pain, which ultimately led to the elective removal of the abutment and implant; the retrieved implant was subsequently examined using multiple advanced analytical techniques to correlate laboratory findings with the patient’s clinical history and symptoms [8]. Later on, the cohort was expanded to include multiple hospitals which forwarded us retrieved implants in a controlled manner for further investigations. In this study, we present the largest multicentre cohort to date, comprising of 21 explanted bone-anchored implants retrieved from 19 patients. By integrating detailed clinical histories with an extensive array of advanced analytical techniques, we aim to correlate clinical outcomes with the tissue profile at the implant interface. This approach is intended to advance translational research in the field of osseointegrated implants.

## Materials and methods

### Implant retrieval and preservation

Following a previously established protocol [8], we applied a structured framework linking clinical histories with cellular, morphological, and microbiological findings using X-ray micro-computed tomography (micro-CT), histology and histomorphometry, immunohistochemistry, and microbiological culturing. Implant retrievals were performed independently by clinicians based solely on clinical indications, including chronic or idiopathic pain, suspected or confirmed infection, mechanical failure, device nonuse, or treatment change, in accordance with local surgical guidelines.

At retrieval, if the abutment was absent, a 5-mm soft-tissue biopsy, including skin and subcutaneous tissue, above the submerged implant was collected in four patients, fixed in 10% neutral buffered formalin (NBF), and paraffin-embedded for histology and immunohistochemistry. Swabs from the implant and/or abutment were placed in transport medium for microbiological culture. The implant with surrounding bone was retrieved *en bloc* using a small-diameter burr, fixed in 10% NBF, and transferred to the Department of Biomaterials biobank (ID513, University of Gothenburg, Sweden). After ethanol dehydration, implant–bone samples were embedded in LR White resin (London Resin Co. Ltd., UK). In some cases, a limited amount of bone remained around the implant because during removal, the drilling trajectory was very close to the implant body. Moreover, soft tissue was not always collected. Clinical data included demographics, comorbidities, implant characteristics, surgical technique, time in situ, and primary indications for retrieval, as well as history of pain, recurrent infection, or inflammation. Pain was extracted retrospectively from routine clinical records as a binary variable (presence/absence) with free-text descriptors such as “chronic,” “idiopathic,” “persistent,” or “recurrent” pain, and was not assessed using a standardized VAS or comparable pain scale.

### Ethics approval

Ethical approvals were handled according to national legislations at the participating clinics. Samples from some centres were obtained as part of clinical investigations with appropriate ethics committee approval (study IDs available in the declarations). Other samples had patient consent for use in academic research per national bioethics policies. Remaining samples were considered waste materials and were exempt from ethical approval. Clinical data were limited to sex, age, comorbidities, implant type, surgical implantation technique, time in situ, reason for explantation, and bacterial species (in cases of diagnosed infection). All samples were coded in the biobank without patient identity access.

### Analytical techniques

#### Microbiological analyses

Bacterial swabs were processed using standard culturing techniques at participating centres. Sterile swab were gently rubbed from the cover screw or the implant top during explantation, and placed in transport medium. DNA extraction was performed followed by standardized molecular techniques (IS-pro™) to identify bacterial species through polymerase chain reaction (PCR) amplification and capillary electrophoresis of 16–23 S rRNA interspacer regions [28].

#### Micro-CT

**Fig. 1:**
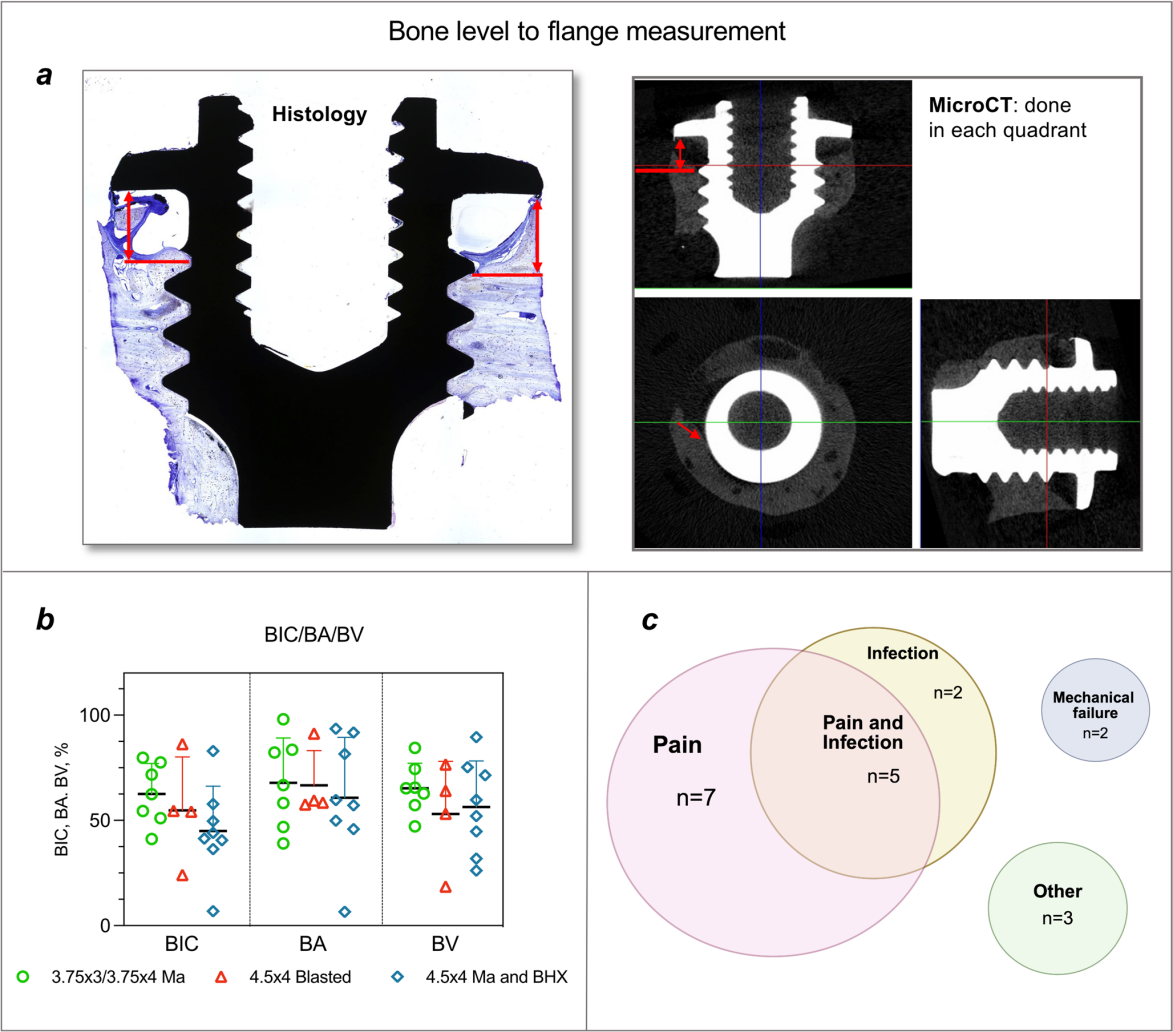
Histological, structural, and clinical characterization of retrieved bone-anchored implants. (**a**) Bone level to flange measurement in histological sections and micro-computed tomography (Micro-CT) reconstructions, performed in two quadrants relative to the orthogonal planes. Red arrows indicate the measured bone crest levels relative to the implant flange. (**b**) Quantitative comparison of bone-to-implant contact (BIC), bone area (BA), and bone volume fraction (BV) among three implant groups: narrow-diameter machined implants 3.75×3 and 3.75×4 Ma (Group 1), 4.5×4 Blasted (Group 2), and wide-diameter machined or laser-ablated implants, 4.5×4 Ma and BHX (Group 3). (**c**) Clinical indications for implant retrieval visualized in a Venn diagram. The majority of cases were associated with pain (*n* = 12), including overlapping cases with confirmed or suspected infection (*n* = 5). Cases of infection with unreported pain (*n* = 2), mechanical failure (*n* = 2), and other indications such as change of treatment/device, or non-use (*n* = 3) are also shown. *Abbreviations: n*,* number of cases; dimensions (e.g.*,*75×4) indicate implant diameter × length in mm; Ma*,* machined surface; BHX*,* Biohelix*,* laser-ablated.*

Bone-implant samples were scanned using Skyscan 1172 micro-CT (Bruker, Kontich, Belgium) at 100 kV, 6.7 μm-resolution, with five images averaged per 0.4° rotation step in a full 360° rotation. Projection images were reconstructed using back-projection, manually aligned along the implant’s long axis, and analysed for bone volume within the threaded region (NRecon, DataViewer, CTAn, CTVox, Bruker, Kontich, Belgium). In summary, a tapered cylindrical volume of interest encompassing the threaded implant portion, with bone volume segmented through manual global thresholding based on morphological characteristics before 3D analysis, as described by Palmquist et al. (2017) [29]. Binary segmented images of the implant, designated regions of interest, and bone within these regions were stored. Bone level relative to implant flange was measured in two quadrants using Dataviewer by aligning orthogonal plane views to assess the bone crest position (Fig. 1a).

### Histology and histomorphometry

Resin embedded samples (implant & per-implant bone) were sectioned transversely (EXAKT^®^ Apparatebau GmbH & Co, Norderstedt, Germany). Ground Sect. (50 μm-thick) were stained (1% toluidine blue, 1% methylene blue, 3% basic fuchsin). Paraffin embedded samples (soft tissue) were sectioned (5 μm-thick; Leica RM 2255, Leica Biosystems Nussloch GmbH, Germany), deparaffinized (xylene), and stained with haematoxylin and eosin. Additional soft tissue sections were stained with Giemsa to visualise bacterial colonies. Qualitative histology was performed with a light microscope (Nikon Eclipse E600, Nikon, Japan). Histomorphometry (Nikon NIS-Elements software, Nikon Instruments Europe BV, The Netherlands) was performed to determine the bone level relative to the implant flange (Fig. 1a), bone-to-implant contact (BIC) and bone area (BA) within the implant threads.

#### Immunohistochemistry

Soft tissue sections were deparaffinized, rehydrated, and rinsed in phosphate-buffered saline (PBS). Heat-induced antigen retrieval was performed (90 °C, 20 min) followed by blocking (5% goat serum in 4% bovine serum albumin, 30 min). Primary antibodies were applied (room temperature, 2 h, dilutions in Online Resource 1, Table S1): iNOS (PA1-036), MRC1 (PA5-82136), CD3 (17617-1-AP), CD20 (PA5-1670), and CGRP (PAS-116153) (Thermo Fisher Scientific, United States). Immunoreactivity was visualized with a horseradish peroxidase (HRP) detection kit (ThermoFisher) and a metal-enhanced diaminobenzidine chromogen. Negative control sections omitted primary antibodies. Whole-slide scans were acquired using Plan Apo 20 × 0.8 objective mounted on a slide scanner (Axioscan 7, Zeiss, Germany) and analyzed in QuPath software (v0.5.1) to quantify immunoreactive cell density.

#### Statistics

Bone-to-implant contact (BIC), bone area (BA), bone volume (BV), and bone level to the flange were compared among the three implant groups using Kruskal–Wallis due to nonnormal data. Statistical significance was set at *p* < 0.05. Values are presented as means ± standard deviation (SD). Analyses were performed using GraphPad Prism (GraphPad Software, USA).

## Results

### Case descriptions

An overview of patient demographics, surgical characteristics and implant time in situ is provided in Table 1. A total of 21 implants were retrieved from 19 patients (mean age 52.6 ± 18.6 years), including 8 males (42.1%), 10 females (52.6%), and one patient with unspecified sex. Smoking status was documented for the majority of patients, with 15 reported as non-smokers (78.9%), one smoker (5.3%), and 3 with unknown status. The average implant time in situ was 72 ± 53.7 months. The surgical techniques included tissue reduction (36.8%), tissue preservation (31.6%), and the minimally invasive Ponto surgery (MIPS, 15.8%) technique. Additionally, one patient (5.3%) had a transcutaneous implant, and for two patients (10.5%) surgical details were unspecified. Comorbidities included diabetes mellitus (21.1%), hypercholesterolemia (5.3%), hypertension (10.6%) (including one post-radiotherapy) and smoking (5.3%), Additional individual conditions included minimal change disease, acoustic neuroma, Down syndrome, autism spectrum disorder, asthma, irritable bowel syndrome, Bland-White-Garland syndrome, gastric bypass surgery, and history of skull fracture. Two patients had no reported medical condition, and several were recorded as non-specified (n.s.).

**Table 1:** Patient demographics and surgical characteristics

Category	Overall
**Patient demographics**	
**Patients**,** n (%)**	19 (100)
Male	8 (42.1)
Female	10 (52.6)
n.s.	1 (5.3)
**Age**,** mean years (SD)**	52.6 (18.6)
**Smokers n (%)**	
No Smoking	15 (78.9)
Smoking	1 (5.3)
n.s.	3 (15.8)
**Surgical technique and time** in situ	
**Surgical technique**,** n (%)**	
Tissue reduction n (%)	7 (36.8)
Tissue preservation n (%)	6 (31.6)
MIPS n (%)	3 (15.8)
Transcutaneous implant n (%)	1 (5.3)
n.s. n (%)	2 (10.5)
**Time** in situ, **months**	72 ± 53.7

*n* number of patients,* n.s *not specified,* SD *standard deviation*, MIPS* minimally invasive Ponto surgery

All patients, except one, had a single bone-anchored hearing implant removed. One patient had three implants retrieved, two of which were used for auricular prosthesis fixation, and one as a BAHS. All but two implants were removed along with surrounding bone tissue, and soft tissue samples were also collected from four patients for further analysis. The time in situ for the implants varied considerably with a mean of 72 months (± 53.7), ranging from 12 to 184 months (1 to over 15 years).

As presented in Fig. 1c, the clinical indications for implant retrieval varied, with chronic pain being the most frequently reported reason, accounting for 63.2% of cases (12 out of 19). In many of these cases, pain was described as idiopathic, persistent and recurrent, despite antibiotic therapy, or occurred without clear clinical signs of infection. For instance, in 58.3% of cases (7 out of 12) where pain was reported as the primary reason for implant removal, no clinical signs of active infection were observed. Suspected or confirmed infection and inflammation were reported in 36.8% of cases (7 out of 19), including five cases with pain and two with no records of pain. These included instances of chronic or recurrent infection, sinus tract formation, and visible signs of inflammation at the skin‒implant interface. Additionally, a history of prior possible infection was reported in 4 patients; however, no clinical signs of infection were observed at the time of retrieval. Mechanical issues, such as complications related to the abutment connection, were documented in 10.5% of cases (2 out of 19). Additionally, 15.8% or patients (3 out of 19) underwent implant removal due to non-use of the device (1 case) and change of treatment/device replacement (2 cases).

### Implant design and osseointegration

**Fig. 2:**
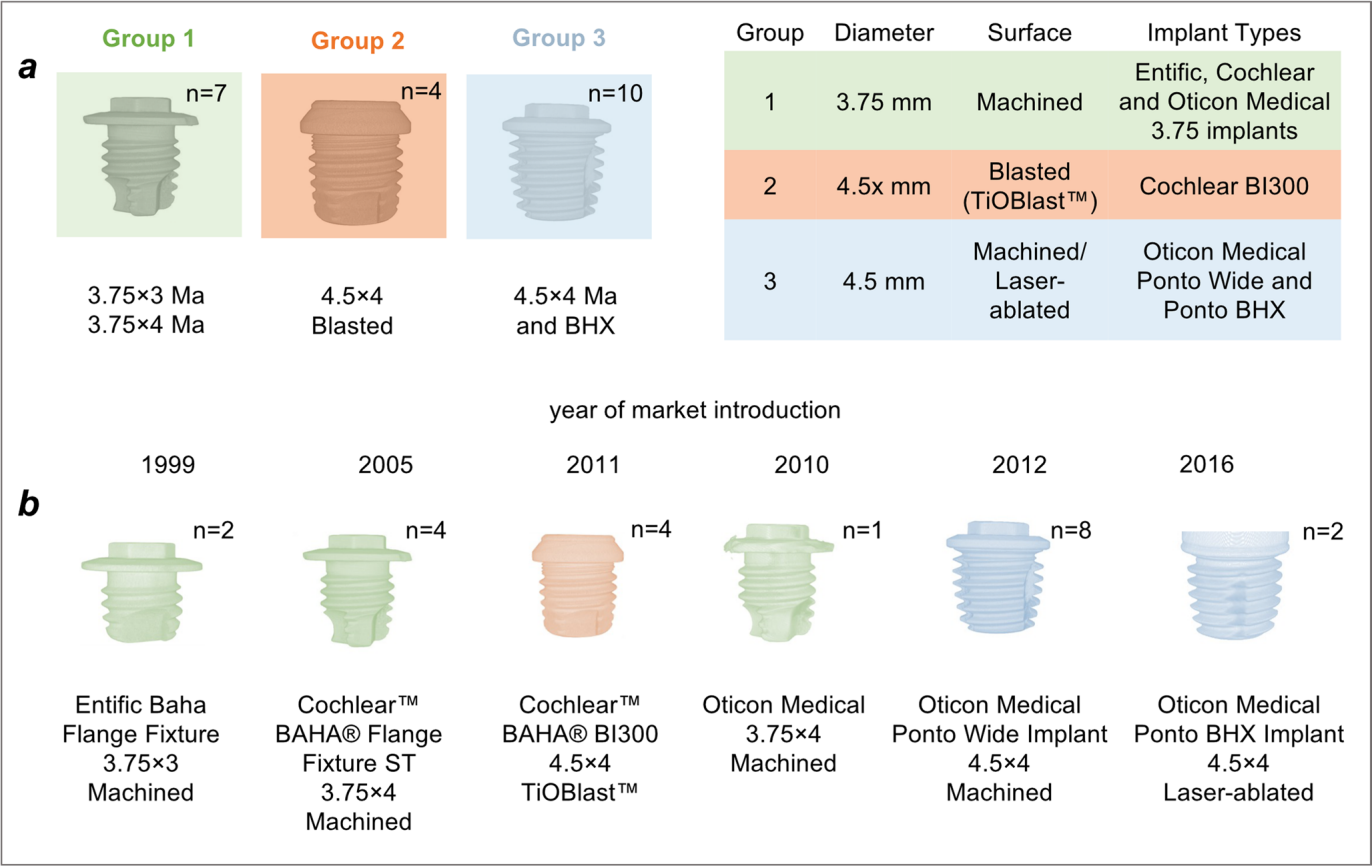
Classification and market introduction timeline of the implant designs included in the study. (**a**) Implants were grouped according to their design, diameter and surface characteristics into three categories: Group 1 – 3.75×3 and 3.75x4 Ma, narrow with machined surface; Group 2 – 4.5×4 Blasted, wide with blasted surface (TiOblast™); Group 3 – 4.5×4 Ma and BHX, wide with machined or BHX (laser-ablated) surface. (**b**) Implant designs listed chronologically by market introduction date. *Abbreviations: n, number of explants; dimensions (e.g., 3.75×4) indicate implant diameter × length in mm; Ma, machined surface; BHX, Biohelix, laser-ablated.*

The micro-CT reconstructions of the implants are displayed in Fig. 2. Taken together, the implant designs analysed in this study span the evolution from early, minimally rough-surfaced fixtures to modern, surface-modified implants. Thus, we assessed how design parameters, such as geometry, diameter, and surface modification, influence osseointegration, clinical performance, and biological responses in percutaneous bone-anchored implants. The implants were grouped according to shared characteristics in diameter, surface treatment, and thread geometry to better capture the functional differences relevant to osseointegration and clinical outcomes, as shown in Fig. 2a.

**Table 2:**
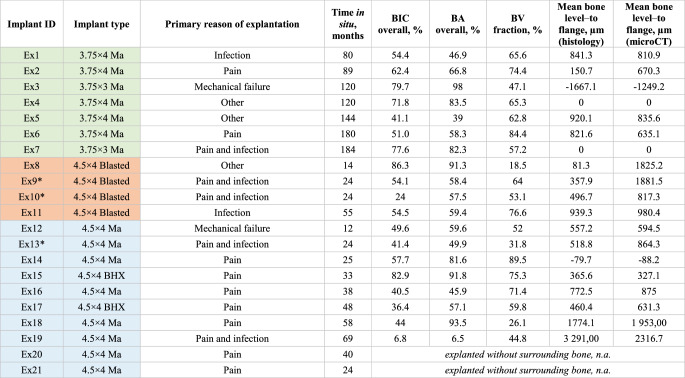
Summary of retrieved implants. Bone-to-implant contact (BIC), bone area (BA), bone volume fraction (BV), and bone level relative to the implant flange from 21 explanted bone-anchored implants. Implants are grouped by design features: Group 1: 3.75×3 and 3.75×4 Ma (green); Group 2: 4.5×4 Blasted (orange); Group 3: 4.5×4 Ma and BHX (blue)

(*) An asterisk indicates implants retrieved from the same patient***. ****Ex* explant number, *Ma* machined surface, *BHX* Biohelix, laser-ablated surface, *BIC* bone-to-implant contact, *BA* bone area,*BV* bone volume, dimensions (e.g., 3.75×4) indicate implant diameter × length in mm, *n.a* not applicable

 Seven implants in the cohort were narrow-diameter implants, 3.75 mm in diameter and 3 mm or 4 mm in length (3.75×3 Ma and 3.75×4 Ma): two Entific Baha Flange Fixtures, four Cochlear™ BAHA® ST Flange Fixtures, and one Oticon Medical implant. All feature relatively fine threads and a smooth titanium surface, reflecting early design principles focused on basic mechanical stability.

 The second group comprises four Cochlear™ BI300 implants (4.5×4 Blasted), which represent a design phase that introduced a moderately roughened surface through TiOblast™ technology. These implants have a diameter of 4.5 mm and a length of 4 mm, and their blasted titanium surface is designed to improve early bone-to-implant contact. Compared to the narrow, machined implants, the 4.5×4 Blasted implants feature deeper threading and a wider platform, providing higher surface area and improved mechanical engagement [15, 30].

 The third and most recent group included 10 wide-diameter implants from Oticon Medical, all of which were 4.5 mm in diameter and 4 mm in length. This group (4.5×4 Ma and 4.5×4 BHX) includes 8 Ponto Wide implants with machined surfaces and 2 Ponto BHX implants featuring a laser-ablated surface. These designs incorporate enhanced thread geometry and surface modifications to optimize both primary stability and long-term osseointegration. The BHX version, in particular, is engineered to stimulate bone remodelling through its increased surface complexity at the microscopic level [17, 31].

 Three implant groups were analyzed: (1) 3.75×4 (n=5) and 3.75×3 Ma (n=2), (2) 4.5×4 Blasted (n=4), and (3) 4.5×4 Ma (n=8) and BHX (n=2). Two 4.5×4 Ma explants (Ex20, Ex21) with insufficient bone were excluded. Bone-to-implant contact (BIC), bone area (BA), bone volume fraction (BV), and bone level to the flange (histology and micro-CT) are presented in Table2. Considerable variability was observed across all groups, with no statistically significant differences (Fig.1b). Most explants had BIC > 40.5%, indicating stable osseointegration; three (Ex10, Ex17, Ex19) were < 37%. Despite lower BIC, Ex10 and Ex17 had relatively high BA (57.5% and 57.1%). Bone level relative to the flange varied widely, with reduced bone beneath the flange observed in several implants across all designs (Online Resource 2, Fig. S1).

 The narrow-diameter 3.75×4 Ma group showed the highest mean BIC (62.6 ± 14.5%) and BA (67.8 ± 21.3%), with BV of 65.3 ± 11.9%. Histological bone level averaged 40.8 ± 932.3 µm, and micro-CT 243.3 ± 747.7 µm, reflecting supracrestal bone in some cases. The 4.5×4 Blasted group (TiOblast™ surface) had mean BIC of 54.7 ± 25.4%, BA of 66.6 ± 16.5%, and BV of 53.0 ± 25.0%. Bone level to flange was 468.8 ± 358.0 µm histologically, but lower by micro-CT (1376.1 ± 555.6 µm), suggesting reduced bone at the collar. The wide-diameter group (4.5×4 Ma and BHX) had mean BIC 44.9 ± 21.4%, BA 60.7 ± 28.7%, and BV 56.3± 21.9%, with bone levels of 957.4 ± 1010.7 µm (histology) and 934.2 ± 808.7 µm (micro-CT), consistent with the other groups.

**Fig. 3:**
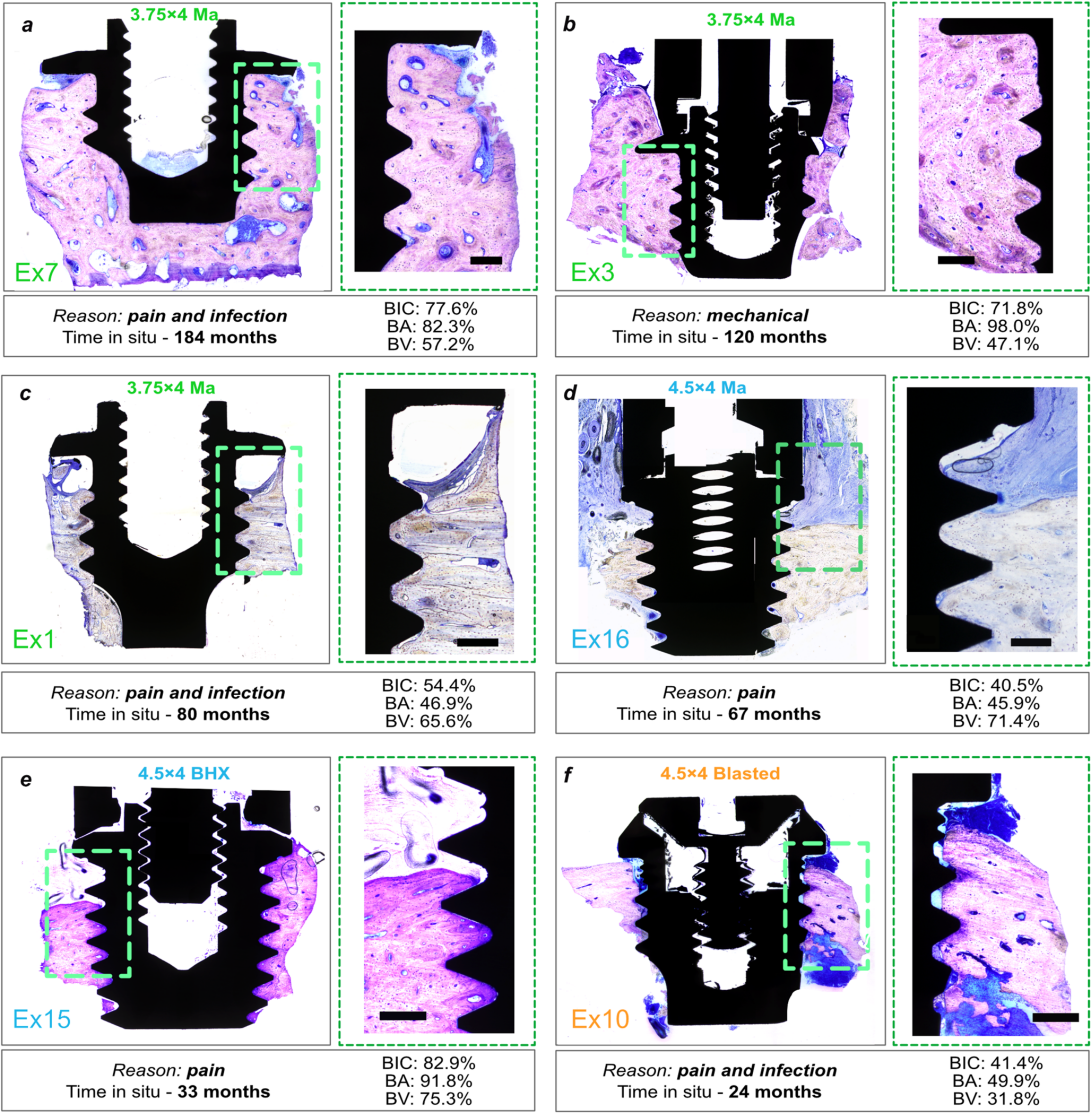
Histological sections of representative explanted bone-anchored implants. Sections stained with toluidine blue and basic fuchsin illustrate bone‒implant interfaces in six representative cases, highlighting variations in bone integration (BIC and BA) and clinical conditions. Each panel shows an overview and a magnified region of interest. Time in situ and reason for explantation are indicated below each sample. Implant groups are indicated by colour: **(a-c)** 3.75×4 Ma (Group 1, green); **(d-e)** 4.5×4 Ma and BHX (Group 3, blue); and **(f)** 4.5×4 Blasted (Group 2, orange). Scale bar indicates 400 µm. *Abbreviations: Ex, explant number; Ma, machined surface; BHX, Biohelix, laser-ablated surface; BIC, bone-to-implant contact; BA, bone area; BV, bone volume; dimensions (e.g., 3.75×4) indicate implant diameter × length in mm.*

 Fig. 3 shows histological sections of six explants ordered by time in situ (>15 to 2 years), illustrating peri-implant bone variability across designs and clinical indications. All had BIC > 40.5%. Older 3.75×4 Ma implants Ex7 and Ex3 (15 and 10 years) had BIC of 77.6% and 71.8%, indicating dense thread contact; in Ex3, excessive bone overgrowth caused abutment connection problems. Ex7 retained osseointegration despite chronic pain and bleeding. Ex1 (3.75×4 Ma, 80 months) and Ex16 (4.5×4 Ma, 67 months) were removed due to pain; both had moderate BIC (54.4%, 40.5%) and lacked bone under the flange, with soft tissue present in Ex16. Ex15 (4.5×4 BHX, 33 months) had high BIC (82.9%) and BV (75.3%) but also lacked bone beneath the flange. Ex10 (4.5×4 Blasted, 24 months) was removed for infection and pain, with low BIC (41.4%) and an atypical interface marked by absence of bone in thread valleys, unlike the uniform bone seen in other implants.

### Microbiology and soft tissue histology

**Fig. 4:**
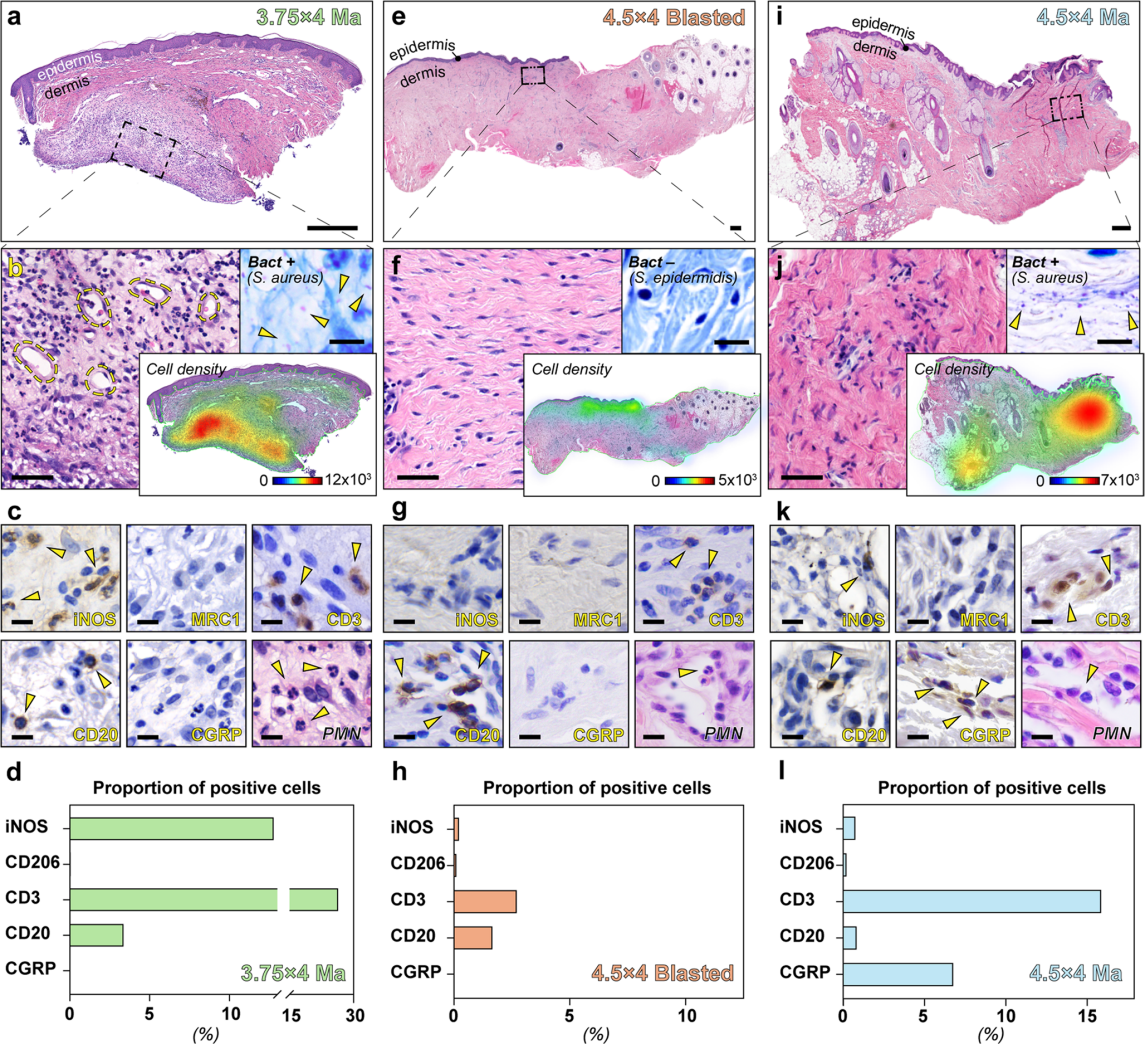
Representative histology and immunohistochemistry of peri-implant soft tissue retrieved at elective explantation due to chronic pain. Panels **a–d**,**e–h **and** i–l **correspond to the 3.75 × 4 Ma (Group 1), 4.5 × 4 Blasted (Group 2), and 4.5 × 4 Ma (Group 3), respectively. **a**,**e**,**i**, Hematoxylin and eosin (H&E) overviews. **b**,**f**,**j**, Higher-magnification H&E highlighting regions with increased cellular density as shown in the pseudo-colorized heatmaps (bottom insets). Yellow dashed circles show vessels. Giemsa staining (top right insets) evidenced bacteria cocci (yellow arrow heads) in the*Staphylococcus aureus*–positive samples (**b**,**j**) but not in the *Staphylococcus epidermidis*–positive specimen (**f**). **c**,**g**,**k**, Immunostaining for iNOS, MRC1, CD3, CD20, CGRP (arrow heads show positive cells). H&E enabled the detection of polynuclear cells suggestive of polymorphonuclear neutrophils (PMNs). **d**,**h**,**l**, Quantification of marker-positive cells (% of total cells) in the regions with increased cellular density (magnified in **b**,**f**, and **j**). Scale bars: **a**,**e**,**i**= 0.5 mm; **b**,**f**,**j**: H&E = 50 um, Giemsa = 10 um; **c**,**g**,**k**= 10 um.* Abbreviation: Ma, machined surface.*

 Soft tissue samples and swabs from a subset of patients were analyzed by histology, immunohistochemistry, and microbiological culturing. Cultures were performed in eight cases, with no bacterial growth in one. *Staphylococcus aureus* was detected in five patients, *S. epidermidis* in two, with *S. capitis* and *Parvimonas micra* co-detected alongside *S. epidermidis* and *S. aureus*, respectively. Four of the five *S. aureus*-colonized patients had chronic pain as the main reason for implant removal, and two showed no clinical signs of infection. Among the seven patients with pain as the sole reason for removal and no clinical signs of infection, soft tissue and microbiological samples were obtained in only two cases, and both showed *S. aureus *growth. Skin-punches including subcutaneous tissue were obtained at explantation from four patients, with confirmed bacterial growth in the three cultured swab-associated samples. Fig. 4 shows three representative cases from groups 3.75 × 4 Ma, 4.5 × 4 Blasted and 4.5 × 4 Ma.

 The 3.75×4 Ma specimen (Fig. 4a–d) was a circular punch harvested ~10 years after implantation and 5 years following abutment removal for recurrent pain. Culture and Giemsa staining confirmed *S. aureus* stromal colonisation with dense, polymorphonuclear neutrophil-rich and highly vascularized infiltrate. Immunohistochemistry revealed abundant iNOS-positive cells (pro-inflammatory macrophages) but no MRC1 expression (pro-regenerative macrophages). Numerous CD3- and CD20-positive mononuclear cells, indicative of T cells and B cells, confirmed the recruitment of cells involved in adaptive immunity.

 The 4.5×4 Blasted sample (Fig. 4e–h) underwent skin breakdown and persistent infection, leading to external transcutaneous explantation while retaining the internal fixture at 5 months post-surgery. Culture revealed *S. epidermidis *and* S. capitis*, but the overlying skin appeared macroscopically intact at with no bacteria detected histologically. The stromal compartment was densely fibrotic with minimal cellular infiltrate and weak iNOS, CD3, and CD20 staining, indicating attenuated inflammatory response.

 The 4.5×4 Ma specimen (Fig. 4i–l) was harvested 25 months after implantation. At month 15, the abutment had been removed for discomfort, leaving the fixture buried for possible re-abutment. The wound was closed without visible inflammation, yet the patient continued to experience intermittent pain motivating elective implant removal. Culture and histology revealed *S. aureus* within the stromal compartment. The inflammatory infiltrate co-localized with bacterial presence, and consisted of neutrophils, iNOS-, CD3- and CD20-positive mononuclear cells but was less pronounced than that in the 3.75×4 Ma specimen.

 Despite pain being the primary indication for explantation, calcitonin gene-related peptide (CGRP), a surrogate marker of nociceptive activation, was detected only in the 4.5×4 Ma specimen.

## Discussion

 This study aimed to employ a multimodal analytical strategy to correlate BAHS patient clinical histories with structural, histological, immunohistochemical, and microbiological characteristics observed in retrieved tissue samples. Pain and infection were the main reasons for implant removal, with infections often associated with the colonization of Staphylococcus species. Notably, *S. aureus* presence was often linked to increased inflammation and reported pain. Despite these complications, nearly all retrieved implants exhibited substantial osseointegration and remained stable in situ. Several previous studies have identified pain as one of the major contributing factors in clinical decision-making for explantation [9, 10, 26, 27, 32–34], often characterized as 'chronic' [9, 27, 34],'intolerable burning sensation' [26], or simply 'persistent' and 'idiopathic' [25, 34]. Notably, 7 out of the 12 patients reporting pain presented no clinical signs of active infection. This observation aligns with a previous case report [8] that indicated unexplained pain as a driver for implant removal but lacked sufficient sample size or analytical resolution to explore its basis comprehensively. In the present study, we had the unique opportunity to examine a subset of cases with indications of pain through a multimodal approach. This included assessment of the bone–implant interface, analysis of soft tissues surrounding the implant, and microbiological swab culture.

 Histological analyses have confirmed that a percutaneous bone-anchored implant alters the “steady-state” immune landscape of the skin. More than three decades ago, Holgers et al. (1995) showed that intact skin over submerged fixtures contain only a sparse surveillance infiltrate of leukocytes [5]. However, breaching the barrier with a titanium abutment approximately doubles this cellularity and skews it toward antigen-presenting cells and B-cell subsets [5, 35]. More recently, we analysed peri-abutment fluid and soft tissue biopsies showing a prolonged inflammatory activity, an upregulation in the local immune response and an ongoing remodelling process around the abutment following implantation of BAHS [36, 37]. In these studies, the gene expression of inflammatory cytokines such as IL-8, IL-1b, and IL-10 and bacteria-related Toll‒like receptors (2 and 4) were higher at 3 and 12 months than at baseline implant surgery.

 Our cohort extends this observation; when *S. aureus* was present, the infiltrate expanded both quantitatively and phenotypically, becoming neutrophil-rich and acquiring a strong iNOS/CD3/CD20 signature. Similarly, shotgun metagenomics of atopic-dermatitis flares links *S. aureus* abundance to clinical severity [38]. The same relationship was found for the 3.75 × 4 Ma and 4.5 × 4 Ma specimens, by combining colonisation with *S. aureus* and pain. In contrast, the 4.5×4 Blasted sample which was colonised by *S. epidermidis *and* S. capitis*, displayed a reduced inflammatory cell infiltration with no associated pain. This finding mirrors reports that the presence of *S. epidermidis* is often not linked to overt clinical symptoms. *S. epidermidis* is a skin barrier-protective commensal that competes with pathogens and primes cutaneous immunity [39]. However, owing to its high strain variability, *S. epidermidis* is capable of rapidly switching to an opportunistic, biofilm-forming pathogen on implant surfaces [40]. Furthermore, prospective swabs of bone-anchored hearing systems have demonstrated that *S. epidermidis* dominates uncomplicated peri-abutment sites, but that it is also enriched during Holgers-grade inflammation [28].

 An important observation in this study was the association between *S. aureus *and pain. Mechanistically, *S. aureus *is capable of direct activation of nociceptors via α-haemolysin, phenol-soluble modulins, and other staphylococcal virulence factors [41, 42]. Recent evidence also suggests an indirect mechanism in which staphylococcal infection the alters the skin immune environment, leading to the activation of the neuropeptide CGRP [43]. Released by the cutaneous sensory fibres, CGRP appears to be important for the cross talk between bacteria and immune system—particularly T lymphocytes—under steady state and pathology. Yet, in our study, only one soft tissue specimen, where *S. aureus* was detected and associated with pain, showed CGRP immunoreactivity. These findings suggest that when the peri-implant niche is dominated by *S. aureus*, nociception is not always CGRP-dependent. Above all, interpreting the pain data remains challenging given that nociception is inherently subjective. Visual-analogue scales offer a numeric anchor but reflect a momentary perception shaped by multiple factors (mood, expectation, etc.) [44]. Unrecorded use of nonsteroidal anti-inflammatory drugs or opioids can further blur the link between microbial load, histology, and reported pain. Therefore, the inconsistent detection of CGRP cannot by itself exclude neurogenic contributions. Future studies should pair multidimensional pain inventories with a broader neuro-immune panel (such as substance P, β-endorphin, nerve growth factor) to map the transmitter landscape that drives peri-implant pain. Notably, in this study, microbiological sampling was primarily performed in cases where infection was clinically suspected. Thus, patients whose primary indication for implant removal was pain, without associated adverse soft tissue events, were not sampled with swabs. In typical cases, involving soft tissue complications, the application of local antibiotic ointment is often a treatment approach [21, 45]. However, if problems such as inflammation, infection and pain persist, oral antibiotics are routinely prescribed [33]. Microbiological sampling is rarely performed to guide treatment decisions targeting specific pathogenic microorganisms. Histologically and structurally, the three implant groups demonstrated variable degrees of osseointegration, with no significant differences identified. Most exhibited considerable bone-to-implant contact despite reported pain and infection. Thus, similar to a previous case study [8], here we confirm that successful osseointegration does not necessarily indicate clinical success. These discrepancies between tissue integration and patient-reported outcomes underscore the complexity of interpreting clinical symptoms in isolation and highlight the importance of integrating histomorphometric and microbiological data. Additionally, the absence of differences in BIC, BA, and BV between implant design groups should be interpreted with caution, as more subtle time- or surgical technique-dependent variations in inflammatory patterns may exist, particularly given the wide variation in time in situ and the heterogeneous implantation techniques in this cohort.

 Notably, all three bone-anchored implant designs demonstrated great variability in bone levels relative to the implant flange. Furthermore, irrespective of design, the results revealed a prominent lack of bone under the flange, similar to previous observations in the literature [4, 9, 10, 12, 14]. In this context, differences between histology- and micro-CT–derived bone levels likely reflect methodological factors rather than simple measurement error: histology provides a high-resolution 2D snapshot that is sensitive to the exact section plane and local heterogeneity around the flange, whereas micro-CT yields a circumferential 3D average that better represents the global bone level, with histology adding complementary local structural detail.

### Strengths and limitations

 Analysing the properties of explanted implant materials and surrounding tissues in humans provides an invaluable source of information, particularly when linked to patient history and treatment outcomes. Implant retrieval is rare; in cases of failed osseointegration or trauma, implants are often lost spontaneously, eliminating the opportunity to preserve the specimens for systematic analysis. Importantly, explantation is typically considered a last resort, undertaken only after all other treatment options have been exhausted. Despite the substantial hearing benefits these devices provide, some patients ultimately elect for retrieval due to persistent complications.

 Thus, this study presents the largest retrospective multicentre case series to date of electively retrieved percutaneous bone-anchored implants, offering a unique opportunity to correlate clinical outcomes with microbiological, histological, and structural tissue-level analyses. The wide range of implant durations in situ—from as short as 12 months to over 15 years—highlights the heterogeneity of the cohort. While this diversity enhances the generalizability of the findings, it also complicates the formulation of broad conclusions. Importantly, patient-specific factors such as comorbidities (e.g., diabetes, prior radiation exposure, smoking, and neurodevelopmental disorders) were not systematically analysed in relation to tissue responses around the implants. The number of implants analysed with complete soft tissue, bone, and microbiological data was constrained by inconsistencies in retrieval procedures across centres. Moreover, pain was retrospectively abstracted from routine clinical documentation (presence/absence) and longitudinal data on pain and inflammation progression or resolution following explantation were not consistently available.

 Looking ahead, these limitations highlight the need for standardized methodologies in future research. This includes incorporating patient self-reported outcomes, developing improved tools for reporting and quantifying pain, and establishing consistent clinical scoring systems to better capture the complexity of patient experiences and peri-implant complications. Further studies employing advanced analytical techniques—such as spatial transcriptomics—could offer insights into molecular pathways involved in pain, including markers of inflammation, nerve presence, and fibrotic remodelling.

## Conclusion

 Pain and infection—frequently associated with colonization by *Staphylococcus* species, particularly *S. aureus* and *S. epidermidis*—emerged as the primary clinical drivers of implant retrieval in this cohort. *S. aureus* was commonly linked to heightened inflammation and reported pain. All explants, except one, demonstrated substantial osseointegration and remained stable in situ, regardless of design features. These findings underscore the complexity of the local immune response at the bone–implant interface, where microbial presence, host tissue reaction, and neural signalling pathways interact in multifactorial ways. Importantly, the analysis of electively retrieved implants provided a unique opportunity to correlate microbiological, histological, and structural findings with patient outcomes. This integrative approach offers valuable diagnostic insights and highlights the potential of retrieved implant analysis to inform more targeted, patient-specific treatment strategies and improve long-term outcomes in patients with percutaneous bone-anchored implants.

## Supplementary Information

Below is the link to the electronic supplementary material.

Supplementary Material 1 (DOCX 2.67 MB)
