# Supplementary material for: Pain and infection are the primary reasons for elective retrieval of bone-anchored implants
Source: Eur Arch Otorhinolaryngol. 2026 Mar 14;283(6):3617–30. doi: 10.1007/s00405-026-10072-8 (PMC13249739; doi:10.1007/s00405-026-10072-8)
Supplement: Supplementary file 1 — Supplementary Material 1 (DOCX 2.67 MB) [file 405_2026_10072_MOESM1_ESM.docx]

**Online Resource 1 Table S1. Primary antibodies and dilutions for immunohistochemistry**

| **Target Protein** | **Host / Isotype** | **Dilution** | **Supplier / Cat. No.** |
| --- | --- | --- | --- |
| Inducible nitric oxide synthase (iNOS) | Rabbit polyclonal IgG | 1:50 | Thermo Fisher, PA1036 |
| Mannose receptor C-type 1 (MRC1) | Rabbit polyclonal IgG | 1:200 | Thermo Fisher, PA5-82136 |
| Cluster of differentiation 3 (CD3) | Rabbit polyclonal IgG | 1:100 | Thermo Fisher, 17617-1-AP |
| Cluster of differentiation 20 (CD20) | Rabbit polyclonal IgG | 1:100 | Thermo Fisher, PA5-16701 |
| Calcitonin gene-related peptide (CGRP) | Rabbit polyclonal IgG | 1:50 | Thermo Fisher, PAS-116153 |

**
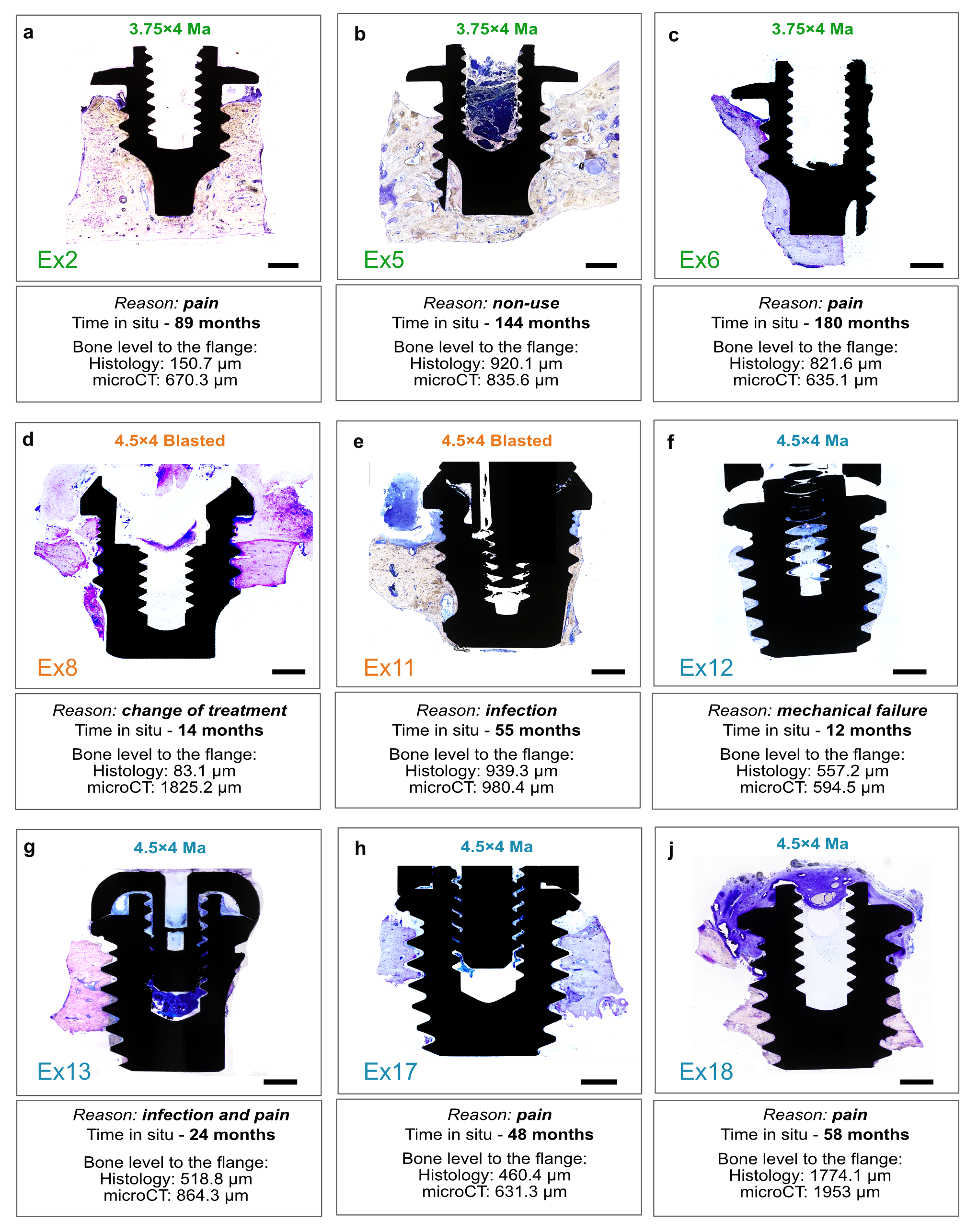
**

**Online Resource 2 FigS1.** **Bone-to-flange level.** Histological sections of electively retrieved bone-anchored implants are shown alongside reasons for retrieval, time *in situ*, and relative distance from bone to the implant flange—measured via histology and micro-CT. All the sections were stained with toluidine blue and basic fuchsin. Panels **(a–c)** (Ex2, Ex5, Ex6) depict 3.75×4 Ma implants (Group 1, green). Panels **(d–f)** (Ex8, Ex11, Ex12) include 4.5×4 Blasted implants (Group 2, orange). Panels **(f–j)** (Ex13, Ex17, Ex18) display 4.5×4 Ma implants (Group 3, blue). Scale bar indicates 1 mm.  *Abbreviations: Ex, explant number; Ma, machined surface; BHX, Biohelix, laser-ablated surface; microCT, microcomputed tomography.*
